# Two attacks of transient global amnesia within a year: a case report

**DOI:** 10.4076/1757-1626-2-6309

**Published:** 2009-08-18

**Authors:** Jan H Schakelaar

**Affiliations:** Huisartsenpraktijk PaladijnenwegPaladijnenweg 30, 3813DJ, AmersfoortThe Netherlands

## Abstract

**Introduction:**

Transient global amnesia is a classical disease. It presents with an abrupt onset of anterograde amnesia. It’s incidence and recurrence rate is low.

**Case presentation:**

In this case report a 54-year old white male is presented with two attacks of loss of memory within a year.

**Conclusion:**

Recurrent transient global amnesia is rare. Recognition and explaining the good nature of the symptoms to the patient and their families is an important task for the doctor.

## Introduction

Transient Global Amnesia (TGA) is a type of memory disorder which often frightens the patients and their families. The usual presentation is a sudden onset of anterograde amnesia: patients aren't able to form new memories. They are disorientated in time and place. At examination there is no neurological symptom or sign. An attack is lasting most of the time less than 10 h and recedes gradually.

TGA generally occurs in persons over 50 years. The incidence is 5-10 per 100.000. The recurrence rate is estimated to be low [1]. Only one of two GP's will ever see a patient with a recurrent TGA.

## Case presentation

Every day I start the day with the Econsults send from the website of our practice. On a morning last February I got this Econsult from one of our patients: “Sometime ago I consulted you for a period of loss of orientation in time. I didn't know my age, which year it was and so on. Last Friday I got the same problem again. I didn't know which year it was; I couldn't grasp it neither on the screen before me or on paper. Temporary loss of a few things from my short memory. It lasted far shorter than the first time. I recognised it immediately. I couldn't go on with my conversation. The last week I suffered from a slight headache”.

The patient who sent me this mail is a 54 old non-smoking white male. He has a slightly elevated blood pressure and uses metoprolol 100 mg once a day. He is 97 kg, 170 cm and uses a few alcoholic drinks only in weekends. In 1997 he was operated (uvula-surgery) for an obstructive sleep apnoea syndrome (OSAS).

He had consulted me last year.

On a Saturday morning, while working with the computer, he at sudden had noticed there was something wrong. Nobody was around. He lost grip of time.

He didn't know if it was before or after the year 2000 and couldn't found out either. Afterwards he thought he had searched the house to find out which day and year it was, may-be found out (paper, agenda, computer, TV) but forgot it immediately. As far as he knew he hadn't suffered from any neurological sign. Slowly his memory had recovered.

On the morning of the Econsult I called him. He told me that this second time it happened in his office. Now there were witnesses. They told him afterwards that he asked repetitive questions about time and date. He has been fully alert. The attack lasted short, about twenty minutes. Nobody had seen any staring or automatisms. There hadn't been any neurological sign or symptom. He hadn't suffered from a head injury the days or weeks before.

Both the attacks were preceded by some psychological stress.

I was a little bit worried after the second attack. Although I had seen two patients with TGA in the past, the recurrence within a year and the headache (afterwards I learned it is a common sign) made me insecure. I phoned a neurologist in the local hospital. She agreed to see the patient. A neurological examination was performed; also a CT-scan was made. Patient was also seen by a cardiologist because the patient sometimes appeared to have palpitations, a fact I didn't knew.

The CT-scan showed no abnormalities. The cardiologist couldn't prove either any abnormality. The neurologist concluded that the symptoms “could very well fit the diagnosis Transient Global Amnesia”. The patient and I were relieved.

## Discussion

The 7 diagnostic criteria for TGA are [2]:

1. Presence of anterograde amnesia.
2. Witnessed account of an observer.
3. No clouding of consciousness or loss of personal identity.
4. Only amnesia and no other cognitive impairment.
5. No focal neurological or epileptic symptoms.
6. No recent history of head trauma or seizures.
7. Resolution of symptoms within 24 h.

The second time all the 7 diagnostic criteria were met. Although there weren't any observers the first time (and therefore the diagnostic criteria were not met) it seems likely it was also an attack of TGA. The key differential diagnosis to be considered are Acute Confusional State (ACS), Transient Epileptic Amnesia (TEA), Transient Ischaemic Attack (TIA), Complex Partial Seizure (CPS) and Psychogenic Amnesia (PA) [2,3].

With ACS there is a marked degree of variability of alertness, often with hallucinations. TEA occurs often at awakening and involves an anterograde and retrograde amnesia. Often there are forms of epileptic activity (staring, automatisms). The same applies to (CPS), in contrary to patients with TGA who are alert and attentive. CPS is often precipitated by an aura. TIA's area very often accompanied by neurological signs (motor and sensory symptoms).

The recurrence rate of TGA seems to be low. In a review of 142 case reports the estimated annual rate of confirmed recurrence was 5.8% [1].

## Conclusion

TGA is one of the classical diseases not often seen by many physicians. It often frightens patients and their families. Recurrence rates are low. Recognising and explaining the good nature of the symptoms, the temporary disturbance of the ability to retain new information without lasting damage, is an important task for the doctor to reassure the patient and their families.

## Patient's perspective

I remember it happened to me on a Saturday morning, around ten. I was at home, alone, so I can't provide observations of others. At the time I was quietly working with one of my computers, in my usual working spot at home, in the attic. In both cases described here, I remember having been agitated about something shortly before, the first time about something at home, the second time about something at work.

I have only a very fragmentary recollection of what happened to me the first time. That first time realisation only came slowly to me (meant relatively: more slowly than at the second occasion) something was wrong. The main thing I remember (?) was wandering aimlessly around the house, and not knowing when I entered my study or the kitchen what I wanted to do there. The ‘event’ lasted for a few hours, I seem to remember about three although I can't point out a specific moment it began or ended.

I do remember the feeling of being lucid and having a very clear mind. I was able (or thought I was able can't really decide that) to recognise having lost my sense of time. I found (even during the ‘event’) I could see a difference with my father, when I witnessed him having a TIA. He kept asking what date it was, without giving any indication he knew what was happening to him. I, on the contrary, found myself very lucid and believed to have a good view of what was happening to me. Whether that was a correct deduction remains to be seen, especially if we look at the observations of some others related to the second time this happened to me.

The second time, the 9th of January 2009, I immediately recognised what was happening to me. (It was recognition of the symptoms as well as a recollection of the previous occurrence). I was a Friday. Again I was quietly at work (no special circumstances), in my office. Since I have my own room I'm not under continuous observation by others.

Also again I felt I was very lucid, especially at the start. At the same time I seemed to realise I wasn't so lucid after all.

I “remember” trying to analyse whether there was more wrong with my memory. Doing that I recalled we had had a farewell party for one of our headmasters the day before. Now I couldn't remember the names of his three children I had met for the first time at that occasion. (Later, after the ‘event’, I was able to recall their names). I now did realise I was 54 years of age, so we had to be at the end of 2008 or the beginning of 2009 (my date of birth is November 6, 1954). I also remember seeing the actual date on the screen left no impression: it didn't help me to regain my sense of time: it was a more or less abstract fact (usually I get a mental image with a certain time or date). I also noticed I completely forgot the date I had just read, the moment I turned away from the screen.

Not being of a clear mind: I did realise something was wrong. When one of our fellow workers, with whom I have to be rather cautious, entered my office for an appointment, I told him I had some minor problems with my memory and asked to postpone our meeting. This we did and he left.

I did have a meeting with another co-worker, with whom I have an easier contact. A week after the incident he told me that the next Monday I had repeated the whole conversation we had on that Friday. I now do remember we had a conversation the Friday of the incident but I still cannot remember what we discussed.

Regarding your question about others observing my behaviour I asked my colleague who happened to come into my office at the time of the occurrence. He told me I had repeated several times the (written above) story of my lost sense of time.

As far as I recall the event lasted about twenty minutes. During the event I remembered being told after the first occurrence I didn't need to worry about it as it was nothing serious. Therefore I was less worried than the first time and tried to actively recognise and analyse what was happening to me. One can well place question marks at the values of my observations if one takes into account the observations of others. If my own observations don't even cover my actual behaviour at the time, what then is the value of what I now remember having observed at the time of the occurrence?

## Abbreviations

ACS: acute confusional state
CPS: complex partial seizure
OSAS: obstructive sleep apnoea syndrome
PA: psychogenic amnesia
TEA: transient epileptic amnesia
TGA: transient global amnesia
TIA: transient ischaemic attack
